# Quality assurance of Cyberknife robotic stereotactic radiosurgery using an angularly independent silicon detector

**DOI:** 10.1002/acm2.12496

**Published:** 2018-11-22

**Authors:** Sultan Fahad Alhujaili, Giordano Biasi, Faisal Alzorkany, Garry Grogan, Muhammed A. Al Kafi, Jonathan Lane, Benjamin Hug, Abdullah H. Aldosari, Sami Alshaikh, Pejman Rowshan Farzad, Martin A. Ebert, Belal Moftah, Anatoly B. Rosenfeld, Marco Petasecca

**Affiliations:** ^1^ Centre for Medical Radiation Physics University of Wollongong Wollongong NSW Australia; ^2^ Radiology and Medical Imaging Department College of Applied Medical Sciences Aljouf University Aljouf Saudi Arabia; ^3^ Biomedical Physics Department, Research Center King Faisal Specialist Hospital and Research Center Riyadh Saudi Arabia; ^4^ Department of Radiation Oncology Sir Charles Gairdner Hospital Nedlands WA Australia; ^5^ Department of Medical Physics and Clinical Engineering Oxford University Hospitals NHS Foundation Trust (Churchill Hospital) Oxford UK; ^6^ Department of Radiation Oncology Sir Charles Gairdner Hospital Perth WA Australia; ^7^ School of Physics and Astrophysics University of Western Australia Perth WA Australia; ^8^ Ministry of Health Riyadh Saudi Arabia

**Keywords:** angular dependence, cyberknife, silicon detector, small field dosimetry, stereotactic radiosurgery

## Abstract

**Purpose:**

The aim of this work was to evaluate the use of an angularly independent silicon detector (edgeless diodes) developed for dosimetry in megavoltage radiotherapy for Cyberknife in a phantom and for patient quality assurance (QA).

**Method:**

The characterization of the edgeless diodes has been performed on Cyberknife with fixed and IRIS collimators. The edgeless diode probes were tested in terms of basic QA parameters such as measurements of tissue‐phantom ratio (TPR), output factor and off‐axis ratio. The measurements were performed in both water and water‐equivalent phantoms. In addition, three patient‐specific plans have been delivered to a lung phantom with and without motion and dose measurements have been performed to verify the ability of the diodes to work as patient‐specific QA devices. The data obtained by the edgeless diodes have been compared to PTW 60016, SN edge, PinPoint ionization chamber, Gafchromic EBT3 film, and treatment planning system (TPS).

**Results:**

The TPR measurement performed by the edgeless diodes show agreement within 2.2% with data obtained with PTW 60016 diode for all the field sizes. Output factor agrees within 2.6% with that measured by SN EDGE diodes corrected for their field size dependence. The beam profiles’ measurements of edgeless diodes match SN EDGE diodes with a measured full width half maximum (FWHM) within 2.3% and penumbra widths within 0.148 mm. Patient‐specific QA measurements demonstrate an agreement within 4.72% in comparison with TPS.

**Conclusion:**

The edgeless diodes have been proved to be an excellent candidate for machine and patient QA for Cyberknife reproducing commercial dosimetry device measurements without need of angular dependence corrections. However, further investigation is required to evaluate the effect of their dose rate dependence on complex brain cancer dose verification.

## 1 INTRODUCTION

Stereotactic radiosurgery (SRS) is a modern radiotherapy technique that employs multiple narrow beams to deliver conformed and precise high radiation dose to the target from different directions in single or few fractions.^1^, ^2^ It requires an accurate target localization and identification which can be achieved by physical stereotactic immobilization devices registering patient to a fixed frame (e.g., Gammaknife) or by imaging‐guided methods (such as Cyberknife Synchrony).^3^ Due to the small beam size and precise conformation of dose distribution, SRS treatment can reduce radiotoxicity to normal tissues and organs at risk and improve the probability of local tumor control.^4^ It is used often for intracranial (brain tumor) and recently extracranial lesions such as spine and breast tumors.

The small treatment volume sizes that are used in SRS introduce several dosimetric challenges for quality assurance (QA) which are not observed in standard conformal radiotherapy. Most predominant challenges are related to the dimensions of the detectors relative to the radiation field size which leads to a volume averaging effect and the fluence perturbation caused by the materials adopted for fabrication of the devices. Perturbation is created due to the variety of stopping power ratios of the materials composing the sensitive volume and surrounding packaging of the detector relative to water and consequently the alteration of the detector response.^5^, ^6^, ^7^, ^8^, ^9^, ^10^, ^11^, ^12^ Due to these effects, the uncertainty in small field dosimetry is significantly higher and errors are notably larger than in dosimetry of traditional radiotherapy field sizes. In nonisocentric radiation delivery modalities, all these effects must be combined with the angular dependence of the dosimetry devices which cannot be easily mitigated using a correction factor based on the relative position of the linac gantry. Ideally, the detectors used for QA in robotic SRS equipment such as Cyberknife should be energy, dose rate, and angular independent. In addition, they should have the ability to obtain high spatial resolution measurement without perturbing the radiation beam.^4^, ^12^, ^13^, ^14^

Although ionization chambers are considered a reference standard in radiotherapy dosimetry,^4^, ^15^, ^16^ the relative large size of the sensitive volume introduces severe volume averaging effects for the smallest field sizes which overestimate the penumbra of the field and underestimate the output factor.^4^, ^17^ Additionally, mini chambers suffer from reduction in their sensitivity and increased noise level due to their small sensitive volume size.^4^ Radiochromic films have been widely used in small field dosimetry because of their near water‐equivalent material and the suitability for measuring dose profiles with high spatial resolution.^18^, ^19^ They are also angularly independent but suffer from lack of reproducibility which depends on processing conditions and procedure. Diamond detectors have been of high interest in small field measurement recently for their near tissue equivalence in a photon beam, high spatial resolution, and real‐time readout.^1^ However, they are expensive and exhibit dose rate dependence^5^, ^12^ and interdevice reproducibility. Silicon diodes are one of the most common detectors adopted for small field dosimetry. The relatively low average ionization energy required to produce an electron–hole pair (3.6 eV) and its density make silicon diodes very sensitive and very small sensitive volumes can be manufactured.^20^ The mass collision stopping power ratio of electrons for silicon–water makes silicon diodes almost completely energy independent for MV range energies.^20^ However, the application of silicon diodes in a small field measurement, especially in nonisocentric noncoplanar and flattering filter free (FFF) modalities like Cyberknife, is limited by directional and dose rate dependence.

The angular dependence of silicon diodes results from their geometry and construction; directionality depends also on the energy of incident beam, field size, and the back scattering from the packaging material creating variations in sensitivity up to 25% with angle of incidence.^21^ There have been several reported solutions to overcome detectors responses anisotropy. One solution has been introduced by Jursinc et al. by adding a thin copper disk to the top side of the diode used in the MapCHECK device which has decreased the angular dependence from ±10% to ±1.25%. However, this solution increased the perturbation of radiation beam due to the addition of the copper material which makes the correction factors depend on the beam energy.^21^, ^22^ Westermark et al. proposed another solution by coupling two diodes back‐to‐back similar to the approach used in MOSFETs.^12^, ^23^The combination of two diodes is found to mitigate the angular dependence to just ±3%, but the double mass of the diodes makes this solution unsuitable for small field dosimetry due to a large beam perturbation.^24^ Several correction factors based on the solutions of directional dependence have been adopted by many research groups and companies for the optimization of commercially available silicon diodes used in QA devices such as the Delta4 (ScandiDos, Uppsala, Sweden), ArcCHECK (SunNuclear, Melbourne, FL, USA), and ion chambers arrays such as I'mRT MatriXX (IBA Dosimetry, Schwarzenbruck, Germany). This solution requires the measurement of the angle of the beam with respect to the detector and applying a correction factor for each angle. This approach is not implemented yet for robotic radiotherapy delivery modalities such as Cyberknife SRS which requires a characterization in almost the whole solid angle.

The Centre for Medical Radiation Physics (CMRP) has proposed a solution to overcome the issue of the angular dependence of silicon diodes by replacing the conventional semiconductor planar structure with a design of the junction close to being a symmetrical three‐dimensional (3D) shape and adopting an innovative diode packaging approach. The technology proposed is called “edgeless” or “active edge” detector. This fabrication technology has been developed by the VTT Technical Research Centre of Finland Micro and Nanoelectronics (Finland) within the framework of the international collaboration MEDIPIX, and its application in radiotherapy dosimetry, in combination with the “drop in” packaging technology, is proposed by CMRP.^21^ The basic characterization of the edgeless detectors for dosimetry in external beam radiotherapy is described in Petasecca et al.^21^

The aim of this study was to evaluate the application of the angularly independent “edgeless” detectors as a QA tool for robotic SRS modalities such as Cyberknife^®^ by testing the diodes for routine dosimetric QA and by delivering three full patient plans to a lung phantom which is stationary or moving with a breathing pattern recorded from four‐dimensional CT for the same patients. In this work, absolute and relative measurements including a field size factor, dose off‐axis profiles, and tissue‐phantom ratio (TPR) have been performed. Measurements were also performed for comparison using PTW 60016, SNC Edge, PinPoint ionization chamber, and Radiochromic EBT3 Films.

## 2 MATERIALS AND METHODS

### 2.A Edgeless detectors

**Figure 1:**
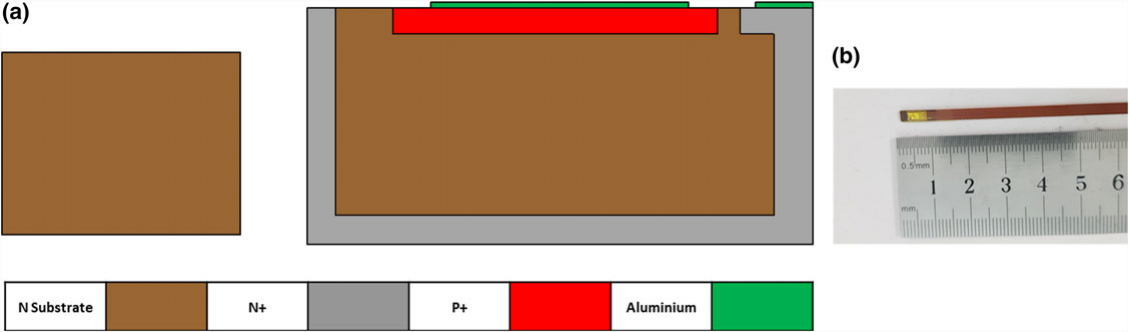
(a) Structure of n‐type edgeless detector of 0.125 mm^3^ volume; (b) Edgeless detector embedded in a Kapton probe using “drop‐in” technology.

The edgeless detectors are fabricated using a lateral implantation technique instead of a standard planar semiconductor fabrication processes. The lateral implantation produces a 3D p–n junction (or ohmic contact) surrounding the die that is leading to full charge collection. Although the edgeless technology allows for processing of both p‐ and n‐type substrates, in this work, the devices adopted are only n‐type, with the top side junction being p + −n and the lateral junction n + −n. The diodes have dimensions of 0.5 × 0.5 × 0.5 mm^3^ [Fig. 1(a)] and are packaged using the “drop‐in” proprietary CMRP technology [Fig. 1(b)]. The packaging is water tight and allows for measurements in a water phantom. The edgeless diodes are readout by a custom‐designed acquisition system based on a commercially available multichannel electrometer named TERA (Tera Foundation, Turin, Italy) which is described in detail by Mazza et al.^25^, ^26^ Additional measurements using stereotactic diodes PTW 60016, SN EDGE and PinPoint ionization chamber PTW 31014, and Gafchromic film EBT3 have been performed for intercomparison and validation of the results obtained with edgeless diodes. The main features of these detectors are summarized in Table 1.

**Table 1:** Properties of the detectors used as reference

Detector	Material	Density (g cm^3^)	Z_eff_	Active volume dimensions	Package material	Reference #
PTW 60016	Silicon	2.33	14	Disk, 0.6 mm radius, 0.03 mm^3^ volume	RW3, epoxy	34
Sun Nuclear EDGE	Silicon	2.33	14	Square, 0.8 × 0.8 mm, 0.03 mm thick, 0.019 mm^3^ volume	Brass	34
Edgeless	Silicon	2.33	14	0.5 mm width, 0.5 mm length, 0.5 mm thick, 0.125 mm^3^ volume	Kapton	21
PTW 31014 pinpoint	Air	0.001	7.64	Cylindrical, 1 mm radius, 5 mm length, 0.015 mm^3^ volume	PMMA, graphite	34

### 2.B CyberKnife^®^ robotic stereotactic radiosurgery systems

CyberKnife is a SRS machine that consists of a portable linear accelerator mounted on an industrial robotic arm (manipulator). By utilizing a set of collimators and a sophisticated imaging‐based tracking system, CyberKnife can produce small, noncoplanar radiation beams and deliver them to a target located near to critical structures. There are two different collimation systems: one system is a collection of fixed collimators (cones) which are manufactured from metallic material with 12 different diameters (from a diameter of 5 to 60 mm). The second system is the Iris^TM^ collimator, a variable aperture diaphragm which adopts 12 tungsten–copper alloy segments arranged into two different banks of six segments rotated approximately 15 degrees each other. By using these segments, the Iris collimator can be shaped into approximately circular shapes with a diameter varying from 5 to 60 mm. The measurements have been performed on two different versions of Cyberknife: G4 and M6. The M6 machine, located at Sir Charles Gardiner Hospital in Perth (Australia), produces a photon beam with dose rate up to approximately 1000 MU min^−1^ while the CyberKnife G4, located at the King Faisal Specialist Hospital and Research Centre in Riyadh (Saudi Arabia), is limited to a maximum dose rate of approximately 800 MU min^−1^. While the basic dosimetric measurements with the edgeless diodes have been performed on the G4 machine, the phantom study measurements were performed using both the M6 and G4 generations Cyberknife.

### 2.C Plastic and water phantoms

Relative dose measurements were performed using medium and large sizes PTW MP3 motorized water tanks (PTW, Freiburg, Germany). Both tanks include three stepper motors which allow the detector to be moved in three different directions. The speed and positioning accuracy of the stepper motors is approximately 50 mm/s and ±0.1 mm, respectively. Both tanks are positioned above an electromechanical lifting carriage to give the ability to adjust the height in respect to the beam source. Solid Water slabs (Best Medical, Nashville, TN, USA) of different thicknesses and 30 × 30 cm^2^ area have also been used.

#### 2.C.1 Timber phantom

**Figure 2:**
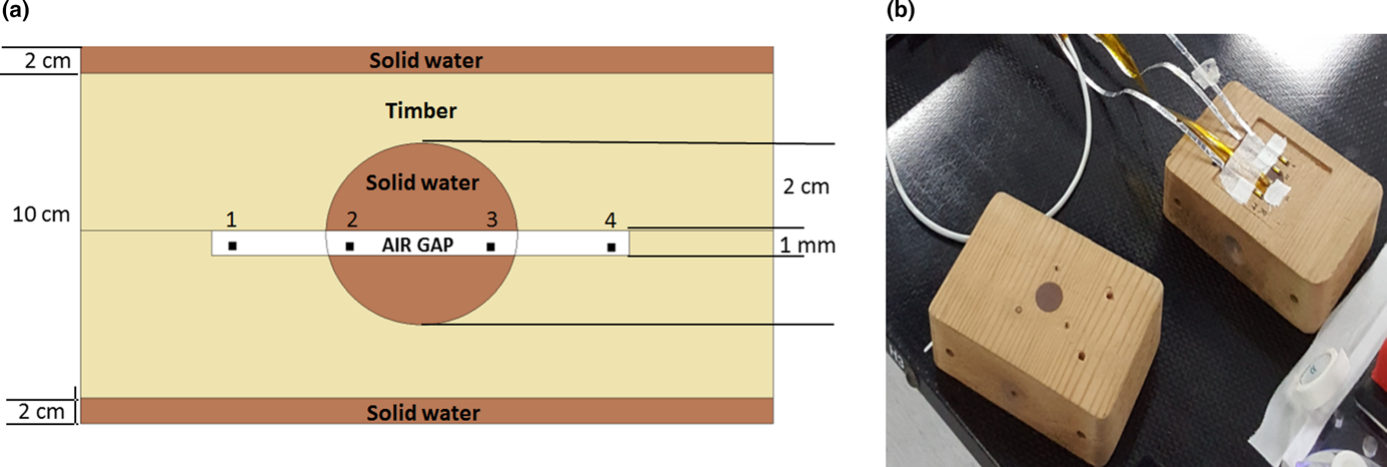
(a) Schematic diagram of the heterogeneous timber phantom; (b) the heterogeneous timber phantom with the detectors placed around the internal lesion. The gold markers are visible as small imperfections of the wood surface in (b).

Cyberknife is also used for clinically suitable lung lesions, particularly when the lesion is in proximity to organs at risk thanks to its capability to track the motion of the target accurately.^27^ In order to test the edgeless detectors for patient‐specific QA, two timber phantoms have been manufactured to mimic a lung with and without an internal lesion. The heterogeneous phantom which presents the internal lesion is composed of two cubic blocks of timber (with a density of approximately 0.3 g/cm^3^) with one hemisphere of solid water in each block positioned at the center of the phantom. The solid water insert mimics a lesion of a diameter of approximately 2 cm inside the lung. The detectors are positioned in between the timber blocks with one hemisphere above and below, to form a spherical lesion with 1 mm gap (Fig. 2). The heterogeneous phantom has been manufactured at the University of Wollongong mechanical workshop and has dimensions of 9.45 × 10 × 14.7 cm^3^ with two slabs of solid water, 2 cm thick above and below the timber blocks to mimic the attenuation from the chest wall muscles and backscattering from the back muscles. In this work, we used also a homogenous version of the timber phantom with the same dimensions and configuration of the heterogeneous phantom but without the internal lesion.

### 2.D Verification of response angular dependence for noncoplanar irradiations

A key characteristic of the edgeless detectors is the angular independence, particularly important in Cyberknife due to its intrinsic noncoplanar radiation delivery. The edgeless detectors have been characterized in terms of angular dependence in cross‐ and in‐plane delivery in Ref. 21 with variation in the response within ±2% for angles between ±90 degree. In this work, we performed also a delivery of the radiation in a plane at 45 degrees between the cross‐ and in‐plane directions. Irradiation has been performed by a Varian Truebeam with 6 MV flattening filter free (FFF) with the couch set at 45 degree and a cylindrical Perspex phantom with the sample placed at isocenter at 15 cm depth. The field size adopted for this test is 10 × 10 cm^2^ collimated using the jaw collimators. We did not perform the angular dependence on Cyberknife because the free‐positioning system of the machine does not allow a fine control of the angle between the beam and the plane of the couch, while the Truebeam alignment system allows for a more accurate positioning of the phantom and control of the gantry around the isocenter.

### 2.E Linearity and calibration factor

Calibration and verification of the response linearity of the edgeless diodes were performed under reference calibration conditions with the Cyberknife head perpendicular to the phantom at source to detector distance (SDD) of 800 mm and using the fixed cone of 60 mm diameter as suggested by the IAEA‐493.^28^ The detectors have been placed at a depth of 1.5 cm and calibrated by irradiating each device in increments of 100 cGy up to a total accumulated dose of 400 cGy. Each irradiation step has been repeated three times to evaluate the repeatability of the measurement.

### 2.F Dose per pulse dependence

Silicon diode sensitivity under linear accelerator beams shows dependency on the instantaneous dose rate (dose per pulse, DPP). Although the dependence to DPP of the edgeless detectors has been established for standard 6 MV‐FF linear accelerators in previous work,^21^ the use with Cyberknife requires further investigation due to the larger DPP delivered and the presence of a large low‐energy photons component in the beam spectrum. In this work, the DPP was investigated in the range of 2.64 × 10^−4^ − 1.67 × 10^−3^ Gy/pulse and obtained by varying the source to surface distance (SSD) from 500 to 1200 mm, with the detectors at a depth of 15 mm in a solid water phantom and collimated by the 60 mm fixed cone. The nominal dose rate was 800 MU min^−1^. The DPP dependency is calculated by normalizing the diodes response to 7.62 × 10^−4^ Gy/pulse, corresponding to the PinPoint ionization chamber (PTW, Freiburg, Germany) response in reference conditions at an SSD of 800 mm.

### 2.G Field size factor measurement

Field size factor is a parameter which must be characterized for each machine and collimation system adopted. The measurement of the field size factor was carried out in a medium size MP3 motorized water tank at the Cyberknife G4. The edgeless diode was attached to a plastic holder allowing it to be remotely controlled for 3D movement in the water phantom with a step resolution of 0.1 mm. The diode was placed at a depth 15 mm and its lateral position was adjusted remotely to obtain maximum signal corresponding to the center of the radiation field from the collimator. The alignment procedure was repeated for each filed size. For filed size, 200 MU was delivered with a dose rate of 800 MU min^−1^. The field size factor of ten different field sizes (5, 7.5, 10, 15, 20, 25, 30, 35, 50, 60 mm) was measured using Iris collimator and at three different SDDs: 650, 800, and 1000 mm. The diode has been aligned using a motorized two‐axis platform. The measurements were repeated three times to estimate the uncertainty and reproducibility of the detector response. The edgeless data were compared to those taken with SN edge (Sun Nuclear, Melbourne, FL, USA).

### 2.H Tissue‐phantom ratio measurement

Tissue‐phantom ratio was measured using a large size (60 × 60 × 60 cm^3^) MP3 motorized water tank (PTW) to allow for more uniform scattering conditions. The diode's positioning and alignment were as described for the field size factor measurements. In each measurement, 200 MU was delivered at SDD of 800 mm and three different field sizes (10, 30, 60 mm) as collimated using Iris collimator. Tissue‐phantom ratio was measured at 13 depth points, from surface to 200 mm. Edgeless diodes measurement has been repeated three times to estimate the uncertainty and the reproducibility of the detector's response and compared with PTW 60016 data measured under the same condition.

### 2.I. Beam profile measurement

Profile measurements were performed with the diode embedded in a solid water phantom equipped with a two‐axis stepper motor stage. After the alignment, performed with the same procedure adopted for OF and TPR measurements, the Cyberknife head was kept static with the radiation beam perpendicular to the phantom surface. The diode was moved across the beam at constant speed (a margin of a few centimeters ensured speed stabilization). The radiation field sizes measured were 5, 10, 30, and 60 mm collimated by Iris collimators at an SDD of 800 mm and a depth in solid water of 15 mm.

### 2.J Patient‐specific QA measurement

In order to assess the performance of the edgeless detectors in patient‐specific QA, the timber phantoms were imaged with Philips Brilliance Big Bore CT Simulator (Philips Electronics N.V., Amsterdam, Netherlands) and Toshiba Aquilion LB scanner. The phantoms were scanned with four diodes inserted for an accurate localization of the sensors and to determine the doses expected in such positions as the calculation of the treatment planning system (TPS). Three fiducial markers were placed in the phantoms to track and correct their position during the treatment with the help of the dual orthogonal x‐ray imaging system. The treatment plans were generated using Multiplan (Accuray Inc., Sunnyvale, CA, USA). The software uses two different dose calculation methods to evaluate the radiation dose absorbed in a medium. One method is Ray Tracing (RTrac) which adopts a classical semi‐analytic method using experimental data such as off‐axis ratio, TPR, and output factor to calculate the dose kernel and the effective path length to correct for heterogeneities.^29^ The second method is Monte Carlo which adopts a virtual source (phase space file of the linac head) to calculate the dose.^29^ Three plans of uniform coverage were created using the RTrac method.

**Figure 3:**
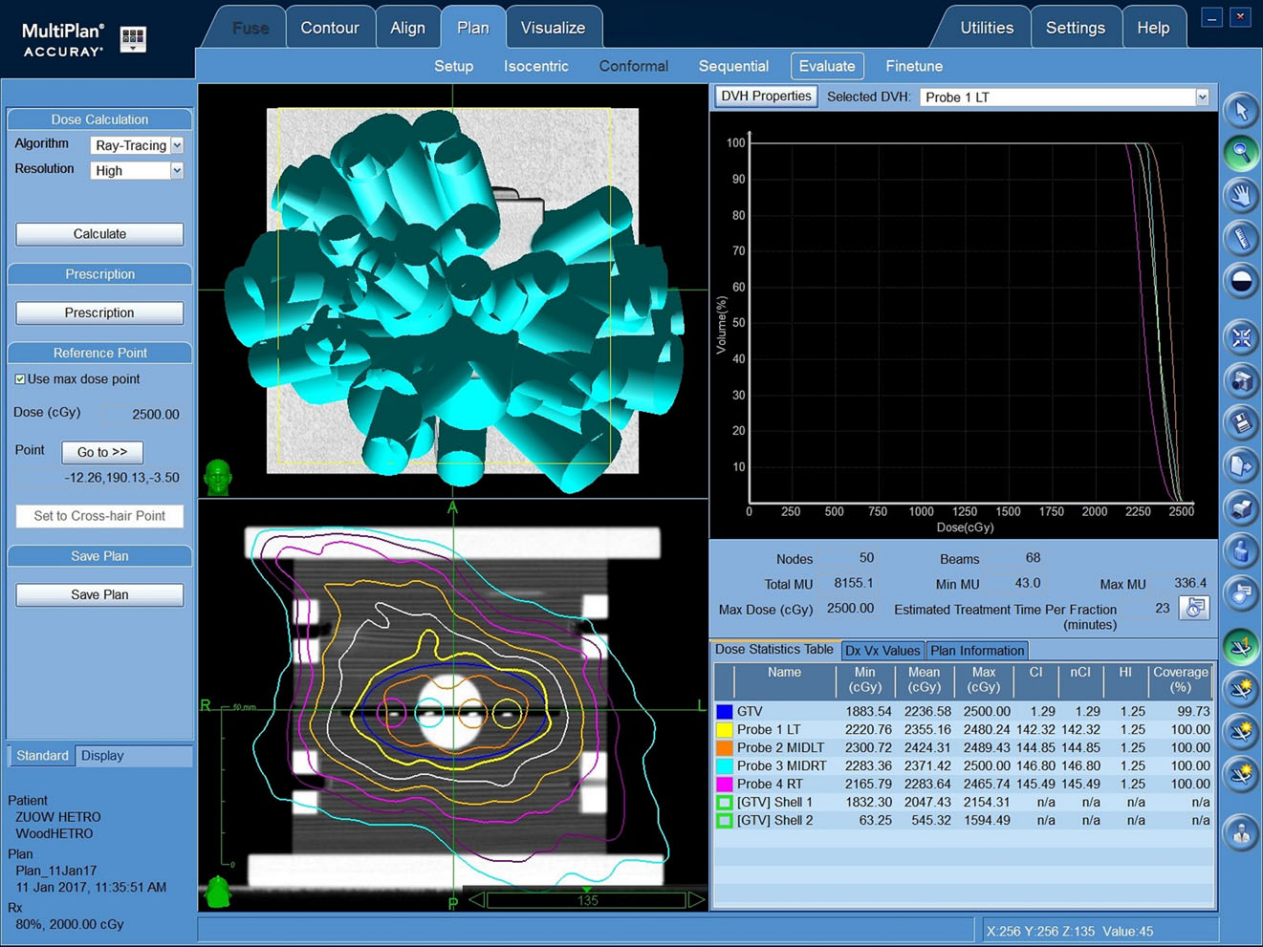
Treatment plan created by Multiplan^®^ for the heterogeneous timber phantom. The diode samples are numbered from 1 to 4.

Plan 1 and Plan 2 were created using the heterogeneous phantom (Fig. 3) and delivered by the CyberKnife G4 and M6, respectively. Plan 3 was created with the homogenous phantom and delivered on Cyberknife M6 with and without a breathing motion simulated by a 3D sinusoidal movement of the phantom. this patterned motion is tracked by the Synchrony Respiratory Motion Tracking System in order to assess the effect of the flashing due to the image‐guided tracking system and of the microphonic noise introduced by the moving platform.

In all plans, the gross tumor volume included the target volume (solid water sphere) and the four diodes. The edgeless detector locations were individually contoured on the CT images of the phantoms in order to evaluate precisely the doses at their locations in the plan and compare them with doses measured experimentally at the same locations in the phantom. At each detector location, average, minimum, and maximum doses were estimated with the TPS.

Figure 3 shows the positions of the detectors inside the heterogeneous phantom: two edgeless diodes were placed inside the spherical solid water target volume whereas the remaining diodes were placed in timber in order to evaluate whether the detector would be able to distinguish the higher dose deposition expected inside the lesion. The plans were incorporated 50 sets of beamlets. Each set (called a node) contains one or more beams which are delivered to the target through unique linac head positions in space. The full set of nodes is called path set which is usually constructed and optimized by the TPS (with no or marginal control from the operator) to deliver the plan. The details of the plans are summarized in Table 2.

**Table 2:** Summary of treatment plans delivered by Cyberknife

Plan No.	Cyberknife model	Phantom	TPS dose at each detector location (Gy) for one fraction				Delivery time per fraction (min)	No. of nodes	No. of beams	Type of collimator
			S_1_	S_2_	S_3_	S_4_				
1	G4	Heterogeneous	6.70	5.93	5.89	5.96	23	50	68	Iris
2	M6	Heterogeneous	5.88	6.70	5.92	5.70	23	50	68	Iris
3	M6	Homogenous	7.30	9.10	9.13	7.92	24	50	68	Iris

### 2.K Patient‐specific QA measurement using EBT3

Gafchromic EBT3 film was used as benchmark for the patient‐specific QA measurements. The film was cut into 7 × 7 cm^2^ pieces and placed inside the phantoms and irradiated under the same irradiation conditions of the edgeless diodes. Each piece was prescanned and scanned 36 h after the irradiation by an Epson XS11000 with 48 bit depth color and a resolution of 72 DPI. In order to minimize the effect of optical nonuniformity, the films were scanned taking care of the orientation and the position on the scanner bed. In order to take into account warming up effects of the scanner, each film has been scanned six times and only the last three images were used to evaluate the optical density. The calibration curve has obtained by irradiating eleven 3 × 3 cm^2^ film cuts from 0 to 1000 MU and scanned using the same protocol. The images of the films have been analyzed using ImageJ version 1.43U (National Institutes of Health, Bethesda, MD, USA).

## 3 RESULTS

### 3.A Linearity and calibration factors

**Figure 4:**
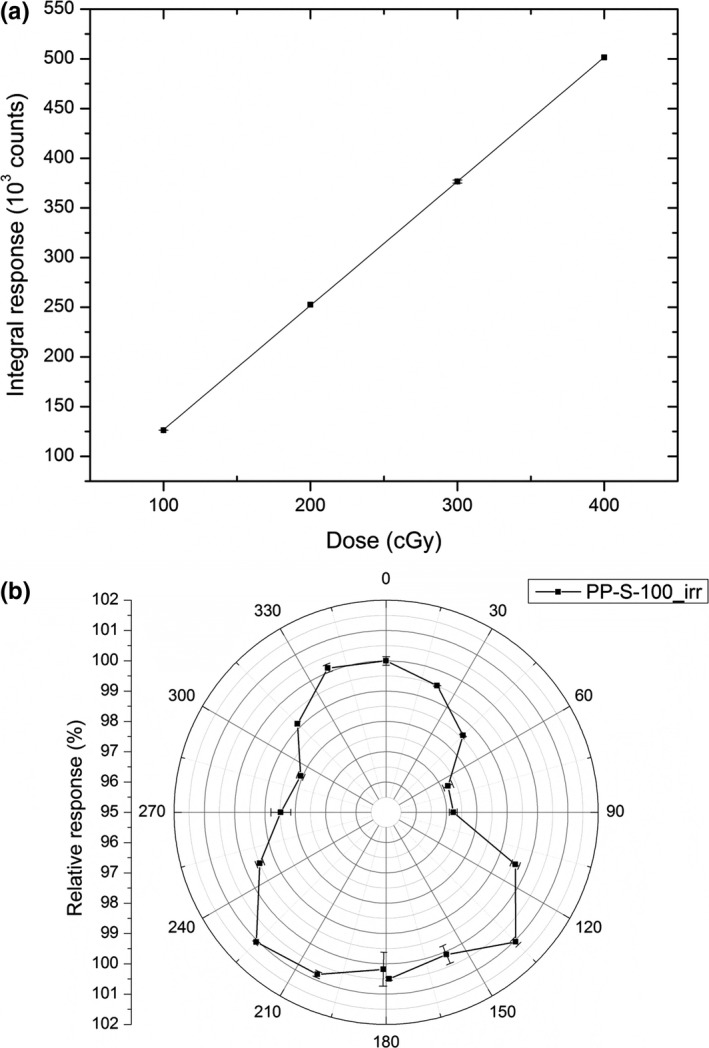
(a) Linearity response of Edgeless diode; (b) angular dependence of the silicon diodes for a noncoplanar irradiation by a Varian True Beam at 6 MV, 10 × 10 cm^2^ field size and couch positioned at 45 degree.

Figure 4(a) shows the dose linearity of the edgeless detector from 100 to 400 with 100 cGy increments. The adjusted regression coefficient *R* ^2^ is 1 and vertical error bars are calculated by two standard deviations over three repetitions. From the slope of the linear fit, the conversion factors from counts to dose for each sample is 1259 ± 6.4 count/cGy (126.4 ± 0.65 pC/cGy).

### 3.B Verification of angular dependence for noncoplanar irradiations

Figure 4(b) shows the response angular dependence of the sample rotating the linac gantry from −180 to +180 degree around a cylindrical phantom. The detector has the connection tail along the axis of the phantom which is placed on the couch. The couch is rotated of 45 degree. The diode shows a variation within ±1.5% also for a noncoplanar beam delivery and in agreement with the results obtained in Ref. 21.

### 3.C Dose per pulse dependence

**Figure 5:**
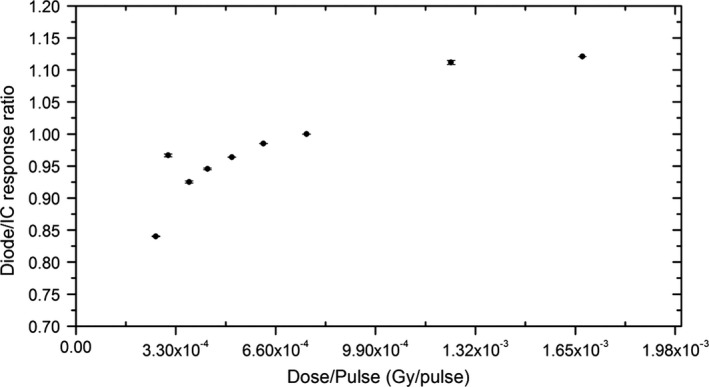
Dose per pulse measurement for edgeless detectors normalized to the measurement by IC at 7.26 × 10^−4^ Gy/pulse corresponding to depth in water of 15 mm, SSD of 800 mm where detector was placed and delivered with the fixed cone of 60 mm diameter. These settings are generally recognized as the reference calibration conditions.

Figure 5 shows the DPP response of the edgeless detectors, normalized to 7.26 × 10^−4^ Gy/pulse representing the response of the IC (MODEL AND BRAND, please) at depth of 15 mm, SSD of 800 mm, and a fixed cone of 60 mm diameter. The error bars representing the uncertainties of the measurements are two standard deviations over three repetitions.

The diodes show a variation in the response of approximately −2% when the DPP is reduced by a factor of 65% of the dose rate at reference calibration conditions (from 7.26 to 4.5 × 10^−4^ Gy/pulse). 65% reduction in the dose rate corresponds to the dose rate variation from a beam collimated by a 60 mm diameter cone to a fixed cone of 5 mm diameter. Such variation suggests that no corrections are required for the response of the edgeless detectors in low‐dose rate conditions. When the SSD decreases, the variation in the response of the detector increases by a factor of approximately +5%, suggesting that for very short SSDs (from 700 to 650 mm), a correction factor should be taken into account to correct for the dose rate dependence of the detector. Applying a correction factor is possible only if the position of the linac head in respect to the target is known. Although this is feasible for machine QA procedures, it may result more complicated for patient‐specific QA.

### 3.D Field size factor

**Figure 6:**
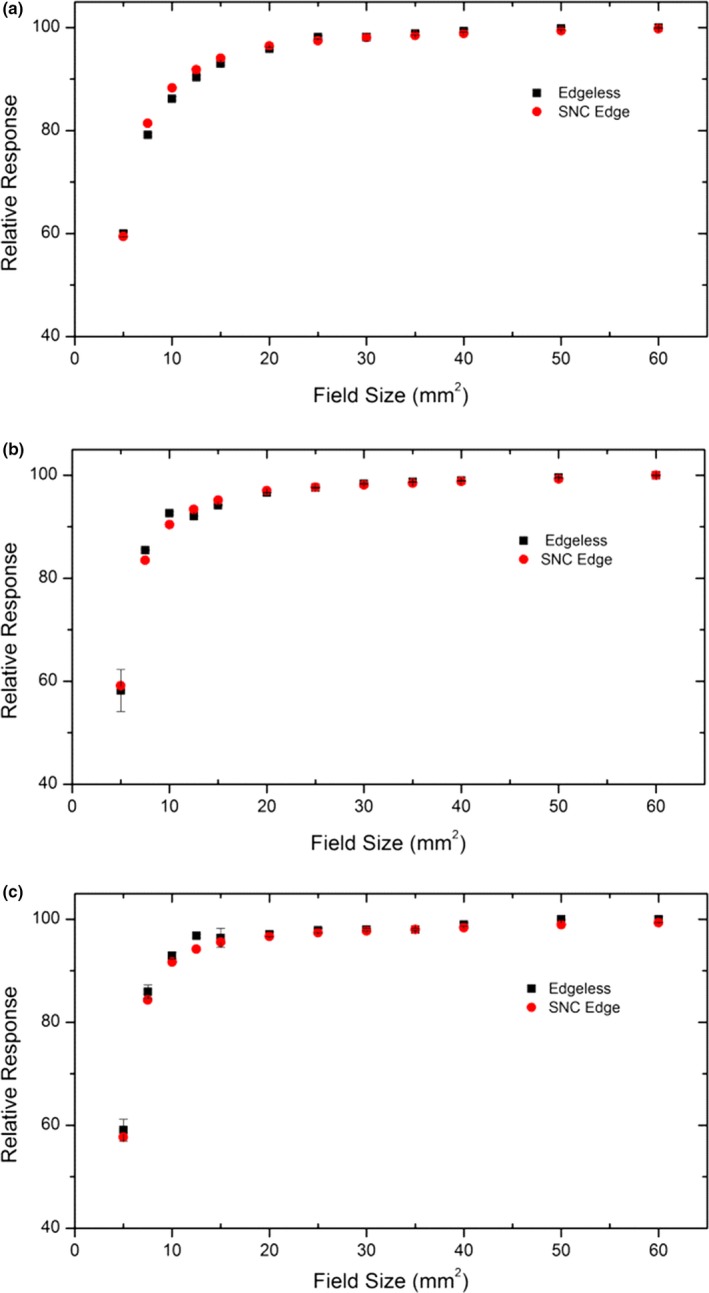
Field size factor for edgeless and SN EDGE diodes for Iris equivalent circular field of 0.5–60 mm at (a) 650 mm, (b) 800 mm, (c) 1000 mm SDD.

Figure 6 show the field size factors measured by the edgeless detector with IRIS collimator. The x‐axis shows the diameter of the equivalent circular field size ranging from 5 to 60 mm at SDD of 650, 800, and 1000 mm. The response of the edgeless diodes has been compared to SNC EDGE diode. The overresponse of the SNC EDGE diodes in the smallest fields has been corrected for by applying the corresponding field correction factors reported by Francescon.^30^, ^31^, ^32^ The edgeless diodes show an agreement with SNC EDGE diodes in the field size range of 25 to 60 mm with discrepancies within ±1%, while at smaller field sizes from 5 to 20 mm, discrepancies do not exceed ±2.6%.

### 3.E Tissue‐phantom ratio

**Figure 7:**
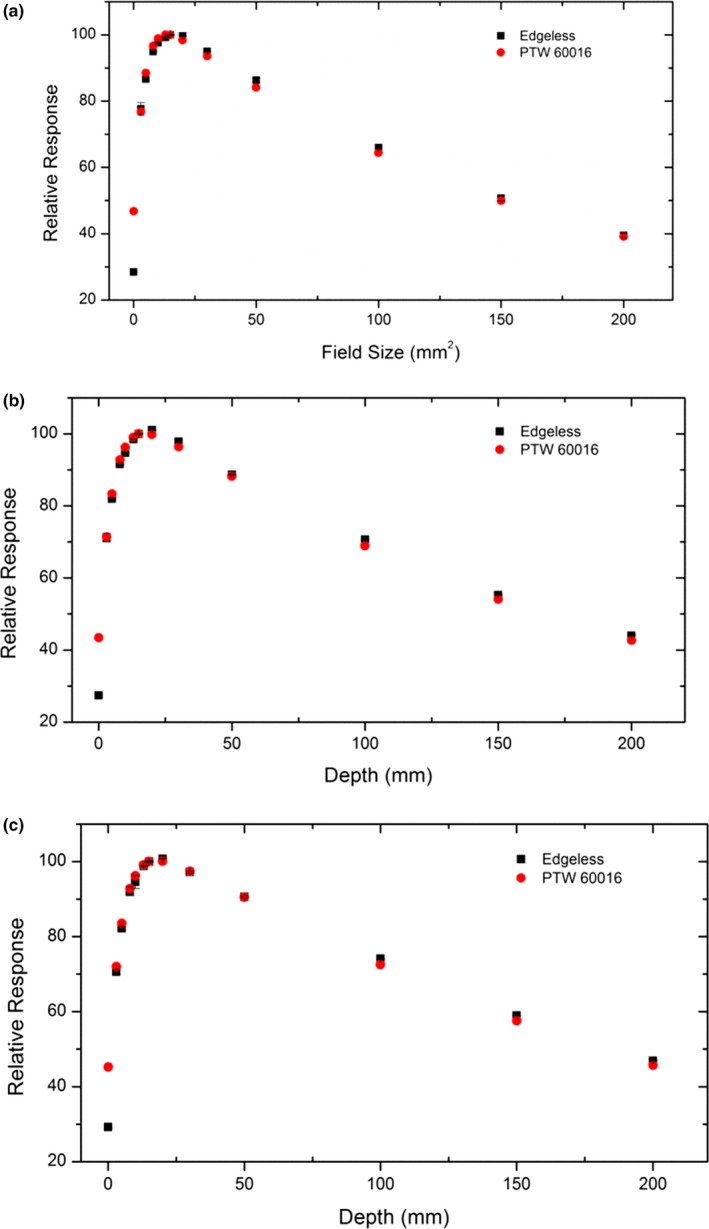
Measured TPR by edgeless and PTW 60016 diodes with 15 mm depth, at 800 SDD mm for Iris circular field size of (a) 10 mm, (b) 30 mm, (c) 60 mm.

Figure 7 shows the comparison of edgeless diode TPR experimental data with PTW 60016 diode's data, obtained with Iris collimator field sizes of 10, 30, and 60 mm diameter. All measurements were performed in a large size water phantom at depths from surface to 200 mm. For this set of measurements, the diodes were attached to the “bird cage”, a tool provided by Accuray Inc., to align them at the center of radiation field and to help maintain the SDD as well. The response of the detectors at each depth is normalized to the measurement taken at 15 mm. Data show an agreement within 2.2% for all the depths except when the detector was placed at the water surface, where the discrepancy is approximately 18.4%. This is due to the minimum buildup created by the packaging of the PTW 60016 which of the order of a few mm of solid water while the edgeless detector is packaged with only 0.07 mm of water equivalent buildup material.

### 3.F Beam profiles measurements

**Figure 8:**
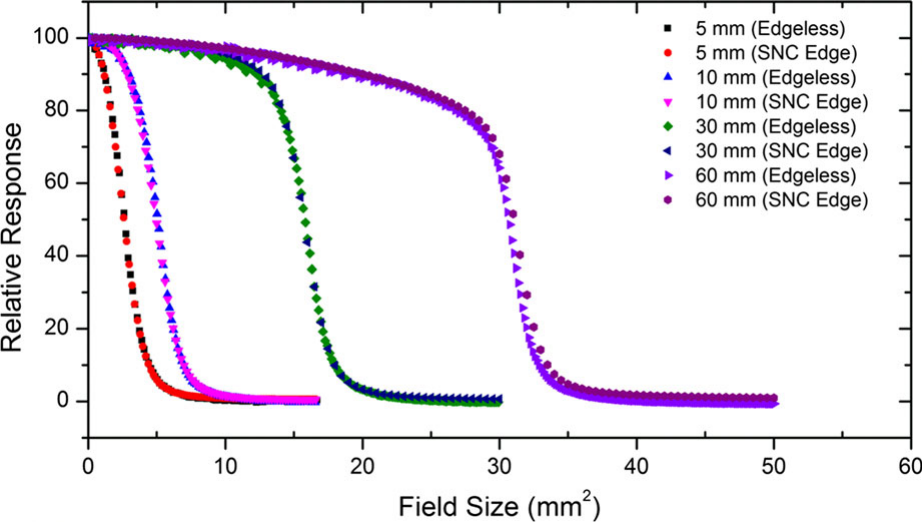
Axis‐off ratios measured by edgeless and SN EDGE diodes at Iris collimator field sizes of 5, 10, 30, and 60 mm.

Figure 8 shows beam profiles measured by edgeless diode and compared to SN EDGE diode. A set of four Iris collimator field sizes are reported with diameter of 5, 10, 30, and 60 mm, measured at a depth of 15 mm at a SDD of 800 mm. The data are normalized to the central axis response. Table 3 shows full width half maximum (FWHM) and penumbra width (80%–20%) of the profiles which have been obtained by using an interpolation‐shape‐preserving fit (with a resolution step of 0.01 mm). Figure 8 and Table 3 show an agreement between the FWHM recorded by the edgeless and the SNC EDGE diodes within 2.3% for all the beam profiles and the discrepancies in penumbra width are within 0.148 mm.

**Table 3:** Experimental results of full width half maximum (FWHM) and 20%–80% penumbra for both edgeless and SN EDGE diodes, measured with Iris collimator field sizes of 5, 10, 30, and 60 mm

Field size (mm)	SN edge		Edgeless		SN edge − edgeless difference		
	FWHM	Penumbra	FWHM	Penumbra	ΔFWHM (%)	ΔPenumbra (%)	ΔPenumbra (mm)
5	5.23	2.08	5.33	2.04	−1.972	1.92	0.040
10	9.92	2.59	10.15	2.44	−2.333	5.72	0.148
30	31.49	2.97	31.60	3.02	−0.377	−1.54	−0.046
60	61.135	5.04	61.37	5.04	−0.39	0.08	0.004

### 3.G Patient‐specific QA measurement

Table 4 summarizes the doses measured for four plans by the edgeless diodes alongside with the doses calculated by the TPS and measured with EBT3 films placed at the same plane where the diodes were positioned. Plan 1 and Plan 2 have been delivered using the Cyberknife G4 to the homogeneous and heterogeneous phantoms, respectively. In this case, the phantoms were static and image guidance was used only to drive the Cyberknife to the target where three fiducial markers have been implanted near the center of the phantom.

**Table 4:** Treatment plans created by Multiplane® for homogeneous and heterogeneous phantoms

Diode #	Delivery mode	TPS (Gy)	Edgeless (Gy)	Film (Gy)	TPS − edgeless difference (%)	Film − edgeless difference (%)	TPS − film difference (%)
Plan 1							
1	Static	5.97	5.74	5.84	3.75	1.59	2.19
2		5.89	5.75	5.99	2.25	4.01	−1.83
3		5.93	5.93	6.00	0.10	1.20	−1.12
4		6.70	5.88	6.00	2.87	1.91	0.97
Plan 2							
1	Static	5.70	5.61	5.72	1.67	2.05	−0.39
2		5.92	5.64	6.11	4.72	7.59	−3.11
3		6.70	5.96	6.08	1.51	1.87	−0.37
4		5.88	5.66	5.84	3.74	3.11	0.65
Plan 3 no motion							
1	Static	7.92	8.27	–	−4.45	–	–
2		9.13	9.48	–	−3.78	–	–
3		9.10	9.17	–	−0.806	–	–
4		7.30	7.12	–	2.396	–	–
Plan 3 motion							
1	Synchrony	7.92	8.13	–	−2.77	–	–
2		9.13	9.17	–	−0.373	–	–
3		9.10	9.32	–	−2.43	–	–
4		7.30	7.13	–	2.34	–	–

In order to evaluate the effect of microphonic noise and possible radiofrequency interference with the edgeless diode response, Plan 3 was delivered by the CyberKnife M6 to the homogeneous phantom in static and dynamic conditions and tracked by the Synchrony Respiratory Motion Tracking System.

The dose measured with the edgeless diodes shows agreement with the TPS data with maximum discrepancy of approximately 4.7%. The maximum discrepancy between film and TPS (Ray tracing) is approximately 3.1% which is smaller than that reported in the literature (Wilcox et al.^33^). The largest discrepancy corresponding to dose measured with the sensor number 2 which is placed across the border of the target (at the edge of the solid water sphere of 2 cm diameter) in the region with the steepest dose gradient where measurement is very sensitive to the positioning of a small volume diode.

The discrepancies recorded by the edgeless detectors in respect to TPS data can also be addressed considering that no correction has been applied to the detector for dose rate dependence. The plans selected for this experiment are all “body path” plans with a source‐to‐target distance (SAD) which varies between 80 and 100 cm. Because of the variation in distance, we have a small variation in the dose rate dependence (approximately 2% for this distance range), which may affect some of the irradiation beams delivered at SAD larger than 80 cm.

## 4 CONCLUSIONS

Real‐time dosimetry and QA of SRS treatments performed by the means of a robotic linear accelerator are challenging due to the small field sizes and nonisocentric beam delivery. In this work, a diode manufactured by an innovative technology named “edgeless” has been tested to estimate the diode's accuracy for small field dosimetry and its use as a real‐time device for patient‐specific QA of SRS treatments delivered by Cyberknife. The combination of the edgeless implantation process with the drop‐in packaging technology has been proven to be an effective solution for fabrication of angularly independent point dosimeters. The dosimetric accuracy of the edgeless detectors has been tested by measuring output linearity, TPR, field size factors, and beam profile at Cyberknife which equipped with both fixed cones and the Iris collimator. The results were compared to commercially available unshielded diodes (PTW 60016 and SN Edge) commonly used in commissioning and routine QA of Cyberknife machines.

The field size factor measured by the edgeless diodes (correction‐free) agrees within 2.6% when compared to the SN EDGE diodes corrected by the appropriate coefficients.

In TPR measurements, the edgeless and PTW 60016 diodes agree within 2.2% for both collimator types.

The measurements of beam profiles have demonstrated an agreement with the reference devices with a discrepancy in FWHM and penumbra width within 2.3% and 0.148 mm, respectively. These encouraging results demonstrate that edgeless diodes exhibit negligible volumetric effect, energy dependence, and dose rate dependence, confirming the reliability of the technology and its maturity to be used as a single point dosimeter for routine dosimetric verifications even in high‐dose gradient region measurements for Cyberknife QA.

Patient plans were also simulated and delivered to a lung phantom with four edgeless diodes placed across the gross target volume. The differences between patient‐specific QA measurements with the edgeless diodes were within 4.72% when compared to TPS, for all the phantom configurations. These preliminary results are limited in terms of type of plan delivered and clinical scenarios adopted but demonstrate that the edgeless diodes are a valuable technology also for patient QA, providing a real‐time dosimetry evaluation also for noncoplanar radiotherapy modalities, without requiring a correction factor for angular dependence, even when organized in an array of multiple single diodes.

## CONFLICTS OF INTEREST

The authors have no relevant conflicts of interest to disclose.
